# EMERGING MODELS OF CARE: THE IMPACT OF PHARMACIST-LED MEDICATION MANAGEMENT IN A TRANSITIONAL CARE PROGRAM

**DOI:** 10.1093/geroni/igz038.2602

**Published:** 2019-11-08

**Authors:** Ebony Andrews, Travonia Brown-Hughes, Ronald Lyon, Shanea D Parker, Brad Lazernick

**Affiliations:** 1 Hampton University, Hampton, Virginia, United States; 2 Senior Services of Southeastern Virginia, Norfolk, Virginia, United States

## Abstract

Transitional care programs have emerged as successful models of care in which to reduce cost and improve health outcomes. However, few transitional care models have directly incorporated the expertise of the pharmacist as an integral member of the care coordination team. Therein lies an inherent limitation of many community-based transitional care programs, the underutilization of pharmacist during all stages of the care transition process. In 2013, the Hampton Roads Care Transitions Project (HRCTP), a partnership between Senior Services of Southeastern Virginia Area Agency on Aging in Norfolk, VA and Hampton University School of Pharmacy, was established. The goal of the HRCTP is to provide medication management services to reduce preventable hospital readmissions for adults 60 years of age and older with targeted diagnoses. Pharmacists work in collaboration with social workers who act as HRCTP care transition coaches. Between May 2017- October 2018, 678 patients were enrolled in the HRCTP. The hospital readmission rate among patients with targeted diagnoses was reduced by 55.3% with an absolute percentage point reduction of 9.9% and estimated savings amount per avoided readmission of $14,400. Patients who participated in the HRCTP showed a 14% increase in the Patient Activation Assessment indicating an improvement in self-managing efficacy. 93% of patients/caregivers indicated they felt more confident in their ability to manage their health, and 91% expressed satisfaction with the program. The program has proven effective in assisting seniors to remain in their home, reducing hospitalizations, promoting health, increasing patient satisfaction, and reducing healthcare cost.

